# Final-year nursing students’ perceptions of humanistic education in nursing: a cross-sectional descriptive study

**DOI:** 10.1186/s12909-024-05377-3

**Published:** 2024-04-09

**Authors:** Jun Zhang, Yangyang Tian

**Affiliations:** Intensive Care Unit, The Second Affiliated Hospital of Zhejiang Chinese Medical University, 310005 Hangzhou, Zhejiang China

**Keywords:** Nursing students, Nursing education, Humanistic education, Perspective

## Abstract

**Background:**

Humanistic education is an important part of nursing education. Final-year nursing students’ perceptions of nursing humanistic education are under-investigated. This study aimed to examined final-year nursing students’ perceptions of nursing humanistic education in both school and hospital.

**Methods:**

This was a cross-sectional descriptive study conducted from May to June 2022 among 107 final-year nursing students with a self-designed questionnaire.

**Results:**

Final-year nursing students recognized the importance of humanistic education, scoring above 4.0 on a 1–5 scale, while their initiative to enhance humanistic qualities was relatively low. Students’ satisfaction with the number of humanities courses offered was only 3.7 ± 0.862. Moreover, 62.6% of students believed there was a need to enhance humanistic environmental development including corridor culture. The “monotonous teaching format” (63.6%) and “teaching methods” (64.5%) have emerged as focal points that students identified as needing attention and improvement.

**Conclusions:**

In the future, nursing humanistic education can be enhanced by increasing the proportion of humanities, improving teaching methods, stimulating students’ learning motivation, and strengthening the construction of humanistic environment.

**Supplementary Information:**

The online version contains supplementary material available at 10.1186/s12909-024-05377-3.

## Introduction

Humanistic care refers to respecting individual differences and addressing the needs of individuals [1]. The famous nursing theorist Jean [2] proposed a significant point in her “Watson’s Human Caring Theory” that humanistic care is the essence and the core of nursing. She believed that caring is the most valuable characteristic offered by the nursing profession. Nursing’s social, moral, and scientific contribution to humankind and society lie in its dedication to human caring ideals on theory, practice, and research [3]. Watson’s theory introduced 10 caring factors. Based on these factors, nurses can provide effective caring that promotes the health and well-being of individuals and families by integrating scientific and humanistic knowledge [4]. Research has also confirmed that healthcare professionals with backgrounds in both the sciences and the humanities outperform those with solely scientific knowledge in terms of practical abilities and qualities [5]. Abundant humanistic emotions and higher levels of humanistic literacy enable healthcare professionals to possess acute insight and profound empathy. This allows them to quickly and accurately assess and meet the physical, mental, and spiritual need of service recipients [6].

The basic meaning of nursing humanistic education is to pass on values rich in humanism and humanistic qualities through teaching by example after paying attention to the meaning of life and its values, promoting the development of empathetic, compassionate, and culturally sensitive nurses [7]. As early as the 1990s, nursing schools in the United States have focused on embodying humanistic education as a key element of educational reform [8]. Subsequently, many medical schools in countries like the United Kingdom, and Japan have incorporated nursing humanistic education into their curriculum, to enhance the humanistic care abilities of nursing staff [9]. The development of nursing humanistic education in China commenced relatively late. In order to rapidly increase the nursing workforce to meet the vast healthcare demands, China has long focused on the cultivation of professionals with adequate nursing knowledge, leading to a neglect of the humanistic spirit [10, 11]. Despite the increasing emphasis on the education of humanistic quality in recent years, overall, it lags behind Western institutions in terms of teaching content, style, and methods [12, 13]. An international cross-cultural study also demonstrated that Chinese nursing students had a lower perceptions of caring compared to their counterparts from Russia, Slovenia, and Croatia [14]. Therefore, stepping up humanistic education in nursing discipline remains an urgent issue in China.

The perceptions and evaluations of the direct recipients of education, namely students, contributes to a comprehensive understanding of the current state of education, which plays an important role in education improvement. In the area of nursing humanistic education, current evidence has centered on experience of innovative teaching strategies [15, 16], study of humanistic literacy levels among faculties/students and their influencing factors [17–20], as well as the significance and impact of humanistic education on students’ competencies [21–24]. However, relatively little was known about the evaluation and feedback from nursing students regarding the existing humanistic education system, particularly within the context of China, a representative country with a shortage of medical resources and developing medical humanities [25, 26].

To fill this gap, we selected final-year nursing students as the subjects to evaluate their perspectives on the current status of humanistic education in both academic and clinical settings. This study may have a positive impact on the exploration of humanistic education reform in China and other countries with similar backgrounds.

## Methods

### Design

This study employed a descriptive cross-sectional survey design with a structured self-administered questionnaire.

### Setting

In China, nursing education predominantly follows a four-year undergraduate, comprising 3 years of school classroom teaching and a fourth year of hospital internship. The curriculum typically consists of 3 parts: public general education courses, basic medical courses, and nursing professional courses. Humanities and social science courses are primarily included in public courses (such as ideology and politics, philosophy, behavioral science, history, literature, and arts) and professional courses (including nursing ethics, nursing psychology, nursing management, nursing education, nursing research, etc.). Additionally, various forms of direct or indirect humanistic education (like lectures, social practices, industry models, etc.) are provided throughout the entirety of undergraduate nursing education.

### Participants

This study recruited the final-year nursing students studying for a baccalaureate degree from a university in eastern China (*n* = 283). They were doing internships at 7 municipal hospitals and 6 university-affiliated hospitals across 7 cities in Zhejiang Province. Inclusion criteria: (1) Undergraduate nursing students who have completed all core courses and are nearing the end of their one-year clinical internships. (2) Voluntary participation in this study. Exclusion criteria: invalid questionnaire. We took a convenience sampling and included all eligible subjects. Eventually, a total of 117 final-year nursing students participated in this study. After removing 10 invalid questionnaires, 107 questionnaires were included in the study, resulting in an effective response rate of 89.2%. Among the participants, there were 67 females (62.6%) and 40 males (37.4%). The age range of the participants was 17–24 years, with an average age of 20.9 ± 2.1 years.

### Data collection

Data were collected using an electronic questionnaire between May 2022 and June 2022. The questionnaire links were generated using the WJX platform (Changsha Ranxing IT Co., Ltd., Changsha, China), a specialized platform for creating online questionnaires. These links include information regarding the study purpose, risks & benefits, confidentiality of data, informed consent statement (completion of the questionnaire by participants was considered as implied informed consent), instructions for the questionnaire, and the questionnaire items. The questionnaire links were distributed by the clinical instructors responsible for nursing students. Researchers had no direct contact with the participants.

### Measurement tool

The tool used in this study was a self-designed questionnaire. We developed the initial draft of the questionnaire based on the research objectives and literature review [27–30]. A clinical preceptor and two nursing professors with extensive teaching experience were invited to assess the appropriateness of the questionnaire items. Following their evaluation, redundant and unnecessary questions were eliminated, and modifications were applied to the descriptions of certain items. The final questionnaire consists of two sections: (1) Students’ cognition and attitudes towards nursing humanistic education; (2) Students’ evaluation and satisfaction with humanistic education in academic and clinical settings (covering curriculum, assessment methods, teaching approaches, and the humanistic environment). Subsequently, a pre-test of the questionnaire was conducted among 30 final-year nursing students (this group was from the Second Affiliated Hospital of Zhejiang Chinese Medical University and was not included in the formal study). The reliability test revealed that the Cronbach’ *α* of the questionnaire was 0.852. In the formal study, the questionnaire demonstrated a Cronbach’ *α* of 0.869 and a KMO value of 0.853 (*P*<0.001), indicating good reliability and validity. Furthermore, the questionnaire mainly employed the 5-point Likert Scale, with scores ranging from 1 to 5 representing “extremely disagree/ dissatisfied” to “extremely agree / satisfied” (3 points for neutrality).

### Statistical analyses

After data collection was completed, the questionnaire system identified data with response times less than 120 s and highly convergent answers as invalid questionnaires. These invalid questionnaires were subsequently removed after verification by the researchers. Data were expressed as mean ± standard deviation (SD) and percentages (%). Data analysis was conducted using SPSS 20.0 (IBM Inc., Armonk, USA).

### Ethical considerations

Due to the low-risk nature of this study in terms of educational observations (cognition, attitudes, and outcomes), it has been exempted from ethical review by the Ethics Committee of the Second Affiliated Hospital of Zhejiang Chinese Medical University in accordance with Chinese ethical review regulations [31]. However, we obtained approval from the leadership of the college and the hospital before data collection. This study adhered to the requirements of the Helsinki Declaration. All participants were told that they could voluntarily chose whether or not to complete the questionnaire, and could withdraw from the study at any time without risk.

## Results

### The cognition and attitude of nursing students towards humanistic education

The results revealed that the level of final-year nursing students’ understanding of concepts such as humanistic education was scored as 4.38 ± 0.709. Students recognized the significance of humanistic education at a high level, with scores surpassing 4.50. However, the actual participation of students in humanities courses was relatively low (4.07 ± 0.832). Furthermore, students rarely engaged in autonomous reading of humanities books (2.22 ± 0.914). More details were in Table 1 and Figure S1.

**Table 1:** The cognition and attitude of nursing students towards humanistic education (*n* = 107)

	Subjects	Mean ± SD
1.	I have heard of concepts like “humanistic care,” “humanistic qualities,” and “humanistic education,” and I understand their meanings.	4.38 ± 0.709
2.	I can comprehend the essence of humanistic care and its significance for nursing.	4.46 ± 0.603
3.	It’s essential to provide nursing students with humanistic education.	4.59 ± 0.513
4.	Studying humanities, such as ethics, social science, and arts, is beneficial for personal growth and a positive worldview.	4.54 ± 0.603
5.	The personality and cultivation of nurses are crucial for the smooth execution of nursing work.	4.58 ± 0.550
6.	In today’s culturally diverse society, it’s crucial for me to continually elevate my level of humanistic qualities.	4.58 ± 0.630
7.	I have a strong interest in studying nursing humanities courses.	4.30 ± 0.717
8.	I frequently attend various lectures or training sessions on humanities and social science organized by school and hospital.	4.07 ± 0.832
9.	I frequently read books in the humanities (five or more books per semester).	2.22 ± 0.914

### Nursing students’ evaluation of nursing humanities education

#### Nursing students’ evaluation of curriculum and assessment of humanities education

In terms of the curriculum, as depicted in Table 2 and Figure S2, students’ satisfaction with the current teaching format of nursing humanities courses was 4.27 ± 0.708, while their satisfaction with the number of humanities courses was only 3.74 ± 0.862. The questionnaire also explored the ideal teaching format of nursing students. For teaching formats in academic settings, the top three preferences were “clinical cases as the main focus, supplemented by theoretical lectures” (40.2%), “promotion of outstanding clinical nursing achievements” (28.0%), and “theoretical lectures as the main focus, supplemented by clinical cases” (22.4%). For teaching formats in clinical setting, the top three preferences were “theoretical lectures as the main focus, supplemented by clinical cases” (50.5%), “emphasizing innovative teaching methods such as discussions, hands-on practice, and scenario simulations” (28.0%), and “clinical cases as the main focus, supplemented by theoretical lectures” (15.9%), respectively.

Final-year nursing students expressed a satisfaction rating of 4.23 ± 0.747 with the current teaching format of nursing humanities courses (Table 2 and Figure S2). Similarly, we investigated the preferred assessment methods for nursing humanistic education among nursing students. The top three assessment methods in school were “open-book examination” (41.1%), “group presentation” (29.9%), and “research report” (12.1%). In hospital, the top three assessment methods were “group presentation” (31.5%), “patients’ evaluation” (25.8%), and “theoretical examination” (16.9%).

**Table 2:** Nursing students’ evaluation of curriculum and assessment of humanistic education (*n* = 107)

Subjects		Mean ± SD
Curriculum		
1.	I think the number of humanities courses offered is reasonable and adequate.	3.74 ± 0.862
2.	Lectures on humanities and social sciences, such as literature, history, philosophy, and art, are frequently held in school and hospital.	4.01 ± 0.807
3.	The humanities and social sciences courses offered are in line with our learning interests.	4.02 ± 0.869
4.	My satisfaction with the current teaching format of nursing humanities courses.	4.27 ± 0.708
Assessment		
1.	Humanistic care is sufficiently weighted in nursing skill assessment or work assessment.	4.11 ± 0.793
2.	My satisfaction with the current assessment methods for the humanities courses.	4.23 ± 0.747

#### Nursing students’ evaluation of the teaching quality and humanistic atmosphere

Final-year nursing students expressed overall satisfaction with the humanistic environment and teaching quality of humanistic education in both schools and hospitals, with scores above 4.10 (Table 3 and Figure S3). Moreover, 49.5% of students identified psychology as the knowledge area that humanities course instructors in the school and hospital should reinforce. Additionally, 62.6% of the students believed that schools and hospitals could strengthen the humanistic environment by fostering features like corridor culture, art sculptures, etc.

**Table 3:** Nursing students’ evaluation of teaching quality and humanistic environment (*n* = 107)

Subjects		Mean ± SD	
		School	Hospital
Teaching quality			
1&2.	Teachers are able to integrate knowledge of humanities and social sciences in their lectures and guide students to respect patients.	4.27 ± 0.634	4.40 ± 0.612
3&4.	My satisfaction with instructors of humanities courses.	4.30 ± 0.644	4.45 ± 0.633
Humanistic environment			
1&2.	Schools/hospitals have a strong humanistic atmosphere with humanistic landscapes, artistic sculptures, corridor culture, etc.	4.17 ± 0.733	4.17 ± 0.720
3&4.	My satisfaction with the atmosphere of humanistic education in the school/hospital.	4.31 ± 0.636	4.27 ± 0.638

#### Nursing students’ overall evaluation of nursing humanities education

The survey showed that “monotonous teaching format” (63.6%) and “teaching methods” (64.5%) emerged as key areas requiring attention and improvement. In addition, 55.1% of nursing students perceived the core of humanistic education to be “cultivation of students’ nursing humanistic qualities” (Table 4).

**Table 4:** Nursing students’ overall evaluation of nursing humanities education (*n* = 107)

Subjects		n	%
1.	The main problem with humanistic education in nursing are (multiple choice):		
	Insufficient emphasis on humanistic education in school/hospital.	40	37.4
	Insufficient attention of humanistic education among students.	50	46.7
	Too few and poorly organized humanities courses.	40	37.4
	Monotonous teaching format in humanistic education.	68	63.6
	Lack of good humanistic atmosphere on campus.	45	42.1
2.	The main improvements that should be made to humanistic education in nursing are (multiple choice):		
	Educational content.	58	54.2
	Teaching methods.	69	64.5
	Assessment methods.	51	47.7
	Curriculum design.	42	39.3
	Social practice.	51	47.7
3.	The focus of humanistic education for nursing students should be:		
	Cultivation of students’ nursing humanistic qualities.	59	55.1
	Broadening students’ knowledge of nursing humanities.	4	3.7
	Cultivation of nursing humanistic thinking.	26	24.3
	Development of nursing humanities skills.	4	3.7
	Cultivation of a sound personality.	14	13.1

## Discussion

### Increasing the weighting of nursing humanities

According to the final-year nursing students, the current number of humanities appeared to be inadequate. It was reported that the content of humanities in the undergraduate nursing education in China constituted approximately 13.7%, significantly less than the 21% in the USA and 17% in Japan [32]. Qian et al. compared the top medical schools in China and the United States. The results showed that the medical humanities in Chinese institutions were significantly less than those in the United States in terms of variety and quantity [12]. The low weighting of humanities subjects not only led to students not attaching importance to them but also hindered the expansion of their horizons. The lack of relevant subjects may restrict the professional development of nursing students, making it difficult for them to adapt to the increasingly complex and diverse medical environment [33, 34]. Consequently, colleges and universities that are in a position should provide students with sufficient choices in humanities as much as possible. Schools with limited teacher resources can consider interdisciplinary and inter-institutional cooperation, as well as strengthening faculty training and exchanges, thus laying a solid foundation for the humanistic education of nursing students.

### Increasing the diversity of teaching methods in humanistic education

From the perspective of nursing students, the primary issue with humanistic education was the monotonous teaching method. As one of the key components in achieving educational objectives, teaching method have a direct impact on students’ learning experiences and outcomes. After interviewing 26 nursing students and nurses, Létourneau et al. [35] found that 17 respondents advocated for the addition or change of pedagogical strategies in humanistic education programs. Storytelling, especially stories shared by patients, was a method that participants believed persuasive and convincing. Moreover, all participants suggested some form of individual or group reflective practice activities. The findings of this study aligned with this. The most favored teaching format among nursing students was “clinical cases as the main focus, supplemented by theoretical lectures,” reflecting nursing students’ eagerness to integrate humanities knowledge into clinical practice. Nursing, as a practice-oriented discipline, placed a significant emphasis on sensory visibility. Hence, integrating practice into the classroom may prove to be an effective approach to cultivating humanistic values [35]. Specifically, employing interactive learning and narrative education methods, such as debates, storytelling, and scenario simulations, can stimulate deep thinking and expression among students, facilitating a better understanding of the complexity of medical humanities [36, 37]. In addition, organizing volunteering such as charitable medical services, nursing home assistance, and rehabilitation for special children not only enhanced students’ clinical skills but also fostered a sense of social responsibility [38].

### Improving students’ initiative in nursing humanities

The findings of this study showed that nursing students were not actively engaged in self-direct learning of humanities knowledge and participation in humanities activities. This may be related to the reasons mentioned above. Medical courses are demanding, with heavy workload of internships, while humanities subjects usually occupy a marginalized position. Students with a utilitarian and selective mindset may easily neglect these less emphasized humanistic courses [39]. Besides, the monotonous teaching format and non-energetic classrooms have also greatly diminished students’ enthusiasm for learning [40]. In addition to enriching humanities subjects and improving teaching strategies, it’s also important to motivate students intrinsically.

Previous research has indicated that intrinsic motivation was a crucial factor influencing learning outcomes [41]. Integrating teaching content with students’ interests can effectively stimulate their motivation for learning [42]. Encouragingly, in this study, 90.3% of nursing students expressed a strong interest in humanistic courses. However, only 83.2% of students felt that existing humanistic courses were somewhat or fully consistent with their interests. Hence, schools could conduct surveys or in-depth interviews with nursing students to identify specific interests and barriers, implementing corresponding improvements [43]. Both schools and hospitals can integrate humanistic education content with online resources accessible to students, diversifying learning approaches and leveraging students’ agency and self-learning abilities. Moreover, based on Richard’s self-determination theory [44], providing appropriate rewards for learning achievements can assist students in developing positive learning attitudes, facilitating a gradual transition into intrinsic motivation.

### Strengthen the construction of humanistic environment in school and hospital

Nursing students suggested strengthening the development of corridor culture, artistic landscapes, and other humanistic environments. This indicated that the humanistic environment in school and hospital, as an implicit pathway to humanistic educational enlightenment, may have been somewhat neglected in the past. Research has demonstrated that immersing students in a robust humanistic atmosphere can empower them to experience the influence of humanism firsthand, leading to changes in their behavioral norms and professional perceptions [45]. For instance, in the orientation training for nursing students, emphasis should be placed on explaining the design principles behind the hospital motto, emblem, and other tangible manifestations of humanistic values in the hospital. This includes outstanding healthcare deed in corridor culture, the stories behind landmark architectural designs and so on [46]. Furthermore, fostering the proactive engagement of all students in landscape design serves not only as a means for individuals to articulate their humanistic qualities but also contributes to an elevated sense of organizational belonging and pride. In conclusion, cultivating an egalitarian, harmonious, and welcoming humanistic atmosphere allows students to naturally develop a humanistic spirit within the educational culture [47].

There were some limitations to this study: the findings focused primarily on the attitudes and opinions of nursing students and did not provide an analysis of the intrinsic influences. Future studies could use a more comprehensive design that incorporates qualitative interviews to increase the depth and applicability of the findings.

## Conclusion

Nursing students, as an educational audience, hold perceptions that help educators better understand their educational needs and expectations, thereby increasing the relevance and appeal of the curriculum. According to this study, final-year nursing students had a positive attitude towards humanistic education, yet there were still some deficiencies in the current state of education. Attaching importance to humanistic education in nursing clinical practice, enriching educational methods, creating a humanistic atmosphere, and stimulating students’ enthusiasm for learning will help to cultivate students’ humanistic qualities. In turn, this will enable nursing students to pay attention to patients’ needs in medical services and better fulfill their medical mission.

### Electronic supplementary material

Below is the link to the electronic supplementary material.

Supplementary Material 1

Supplementary Material 2

Supplementary Material 3

Supplementary Material 4

## Data Availability

Data are available upon reasonable request from the corresponding author.

## References

[1] Newbanks RS, Rieg LS, Schaefer B (2018). What is caring in nursing? Sorting out humanistic and christian perspectives. J Christ Nurs.

[2] Gunawan J, Aungsuroch Y, Watson J, Marzilli C (2022). Nursing administration: Watson’s theory of human caring. Nurs Sci Q.

[3] Sourial S (1996). An analysis and evaluation of Watson’s theory of human care. J Adv Nurs.

[4] Wei H, Watson J (2019). Healthcare interprofessional team members’ perspectives on human caring: a directed content analysis study. Int J Nurs Sci.

[5] Wang Y, Han T, Han G, Zheng Y (2023). The relationship among nurse leaders’ Humanistic Care Behavior, nurses’ Professional Identity, and Psychological Security. Am J Health Behav.

[6] Liu X, Li C, Yan X, Shi B (2022). Psychological capital has a positive correlation with humanistic care ability among nurses. Front Psychol.

[7] Chen L, Zhang J, Zhu Y, Shan J, Zeng L (2023). Exploration and practice of humanistic education for medical students based on volunteerism. Med Educ Online.

[8] Dellasega C, Milone-Nuzzo P, Curci KM, Ballard JO, Kirch DG (2007). The humanities interface of nursing and medicine. J Prof Nurs.

[9] Martimianakis MA, Michalec B, Lam J, Cartmill C, Taylor JS, Hafferty FW (2015). Humanism, the hidden curriculum, and Educational Reform: a scoping review and thematic analysis. Acad Med.

[10] Hou J, Michaud C, Li Z, Dong Z, Sun B, Zhang J (2014). Transformation of the education of health professionals in China: progress and challenges. Lancet.

[11] Wei L, Goez H, Hillier T, Brett-MacLean P. A Visiting Professorship in Undergraduate Medical Education at the University of Alberta: Reflections on possibilities for medical humanities in China, and elsewhere., MedEdPublish. (2016). 2020;9:190. 10.15694/mep.2020.000190.1. 10.15694/mep.2020.000190.1 PMC10699406 38073836

[12] Qian Y, Han Q, Yuan W, Fan C (2018). Insights into medical humanities education in China and the West. J Int Med Res.

[13] Song P, Tang W (2017). Emphasizing humanities in medical education: promoting the integration of medical scientific spirit and medical humanistic spirit. Biosci Trends.

[14] Pajnkihar M, Kocbek P, Musovic K, Tao Y, Kasimovskaya N, Stiglic G (2020). An international cross-cultural study of nursing students’ perceptions of caring. Nurse Educ Today.

[15] Guo K, Luo T, Zhou LH, Xu D, Zhong G, Wang H (2020). Cultivation of humanistic values in medical education through anatomy pedagogy and gratitude ceremony for body donors. BMC Med Educ.

[16] Xue M, Sun H, Xue J, Zhou J, Qu J, Ji S (2023). Narrative medicine as a teaching strategy for nursing students to developing professionalism, empathy and humanistic caring ability: a randomized controlled trial. BMC Med Educ.

[17] Wang Y, Zhang X, Xie Q, Zhou H, Cheng L (2022). Humanistic caring ability of midwifery students in China and its associated factors: a multi-centre cross-sectional study. Nurse Educ Today.

[18] Korkmaz Dogdu A, Aktas K, Dursun Ergezen F, Bozkurt SA, Ergezen Y, Kol E (2022). The empathy level and caring behaviors perceptions of nursing students: a cross-sectional and correlational study. Perspect Psychiatr Care.

[19] Lina M, Qin G, Yang L (2022). Mediating effects of emotional intelligence on the relationship between empathy and humanistic care ability in nursing students: a cross-sectional descriptive study. Med (Baltim).

[20] Jia-Ru J, Yan-Xue Z, Wen-Nv H (2022). Empathy ability of nursing students: a systematic review and meta-analysis. Med (Baltim).

[21] Ng LK (2020). The perceived importance of soft (service) skills in nursing care: a research study. Nurse Educ Today.

[22] Martins V, Santos C, Duarte I (2020). Bioethics education and the development of nursing students’ moral competence. Nurse Educ Today.

[23] Byma EA, Lycette L (2023). An integrative review of humanities-based activities in baccalaureate nursing education. Nurse Educ Pract.

[24] Letourneau D, Goudreau J, Cara C (2021). Humanistic caring, a nursing competency: modelling a metamorphosis from students to accomplished nurses. Scand J Caring Sci.

[25] Lu H, Kitt-Lewis E (2018). Pedagogical differences: a comparative reflection between American and Chinese nursing education. Nurse Educ Today.

[26] Organization for Economic Cooperation and Development. OECD Data of Nurses Per 1000 Inhabitants. [Internet]. 2022 [cited 2024 March 25]. https://data.oecd.org/healthres/nurses.htm.

[27] Uttl B, Smibert D (2017). Student evaluations of teaching: teaching quantitative courses can be hazardous to one’s career. PeerJ.

[28] Gong J, Ruan M, Yang W, Peng M, Wang Z, Ouyang L (2021). Application of blended learning approach in clinical skills to stimulate active learning attitudes and improve clinical practice among medical students. PeerJ.

[29] Lengetti E, Cantrell MA, DellaCroce N, Diewald L, Mensinger JL, Shenkman R (2021). Learning environment and evidence among professionals and students satisfaction (LEAPS), experienced during the COVID-19 pandemic. Teach Learn Nurs.

[30] Liang S, Zhai H, Bu M, Yang H (2021). A survey of humanities perceptions and the need for humanities education in graduate nursing degree programs. J Nurs Sci.

[31] National Health Commission. Approaches to ethical review of research in the life sciences and medicine involving human beings. [Internet]. 2023 [cited 2024 January 14]. https://www.gov.cn/zhengce/zhengceku/2023-02/28/content_5743658.htm.

[32] Li Z, Zhu L. J Z. Current status and progress of nursing education research on humanistic care. Policy Sci Consult. 2021:130–31.

[33] O’Neill D, Jenkins E, Mawhinney R, Cosgrave E, O’Mahony S, Guest C (2016). Rethinking the medical in the medical humanities. Med Humanit.

[34] Macnaughton J (2023). Does medical humanities matter? The challenge of COVID-19. Med Humanit.

[35] Letourneau D, Goudreau J, Cara C (2022). Nursing students and nurses’ recommendations aiming at improving the development of the Humanistic Caring Competency. Can J Nurs Res.

[36] Yuan J, Zeng X, Cheng Y, Lan H, Cao K, Xiao S (2023). Narrative medicine in clinical internship teaching practice. Med Educ Online.

[37] Dias R, Robinson K, Poirier P (2023). The Effect of Simulation on nursing student perceptions of readiness to provide end-of-Life Care. J Hosp Palliat Nurs.

[38] Badger K, Morrice R, Buckeldee O, Cotton N, Hunukumbure D, Mitchell O (2022). More than just a medical student: a mixed methods exploration of a structured volunteering programme for undergraduate medical students. BMC Med Educ.

[39] Li F, Ning L, Li S, Fu Y, Wang Y, Deng Q (2023). Latent profiles of nursing students’ professional identity and their relationship with stress and coping styles during clinical practicum. Nurse Educ Pract.

[40] Pitcher C, Prasad A, Marchalik D, Groninger H, Krishnan L, Pottash M (2022). A pilot study to Understand the Role of Medical Humanities in Medical Education. Med Sci Educ.

[41] Held T, Hascher T (2023). Stability and change of secondary school students’ motivation profiles in mathematics: effects of a student intervention. J Sch Psychol.

[42] Pelaccia T, Viau R (2017). Motivation in medical education(). Med Teach.

[43] Zhu Y, Liu G, Shen Y, Wang J, Lu M, Wang J (2022). Humanistic nursing care for patients in low-resourced clinical settings from students’ perspectives: a participatory qualitative study. Int J Environ Res Public Health.

[44] Ganotice FA, Chan KMK, Chan SL, Chan SSC, Fan KKH, Lam MPS (2023). Applying motivational framework in medical education: a self-determination theory perspectives. Med Educ Online.

[45] Rabinowitz DG (2021). On the arts and humanities in medical education. Philos Ethics Humanit Med.

[46] Deng X, Ye M, Li W, Chen S, Guo J, Zhu J (2023). Development of a humanistic care digital storytelling programme for intensive care unit nursing students: feasibility and satisfaction analysis. Nurse Educ Today.

[47] Wang CXY, Pavlova A, Boggiss AL, O’Callaghan A, Consedine NS (2023). Predictors of Medical Students’ Compassion and related constructs: a systematic review. Teach Learn Med.

[48] National Health Commission. Approaches to ethical review of research in the life sciences and medicine involving human beings. [Internet]. [14 Jan]. Available from: National Health Commission.

