# Supplementary material for: Final-year nursing students’ perceptions of humanistic education in nursing: a cross-sectional descriptive study
Source: BMC Med Educ. 2024 Apr 9;24:392. doi: 10.1186/s12909-024-05377-3 (PMC11005204; doi:10.1186/s12909-024-05377-3)
Supplement: Supplementary file 1 — Supplementary Material 1 [file 12909_2024_5377_MOESM1_ESM.docx]

Supplementary material 2: Figure S1. The cognition and attitude of nursing students towards humanistic education (n=107). Supplementary material 3: Figure S2. Nursing students’ evaluation of curriculum and assessment of humanistic education (n=107). Supplementary material 4: Figure S3. Nursing students’ evaluation of teaching quality and humanistic environment (n=107).
