# Supplementary figures and images for: Final-year nursing students’ perceptions of humanistic education in nursing: a cross-sectional descriptive study
Source: BMC Med Educ. 2024 Apr 9;24:392. doi: 10.1186/s12909-024-05377-3 (PMC11005204; doi:10.1186/s12909-024-05377-3)

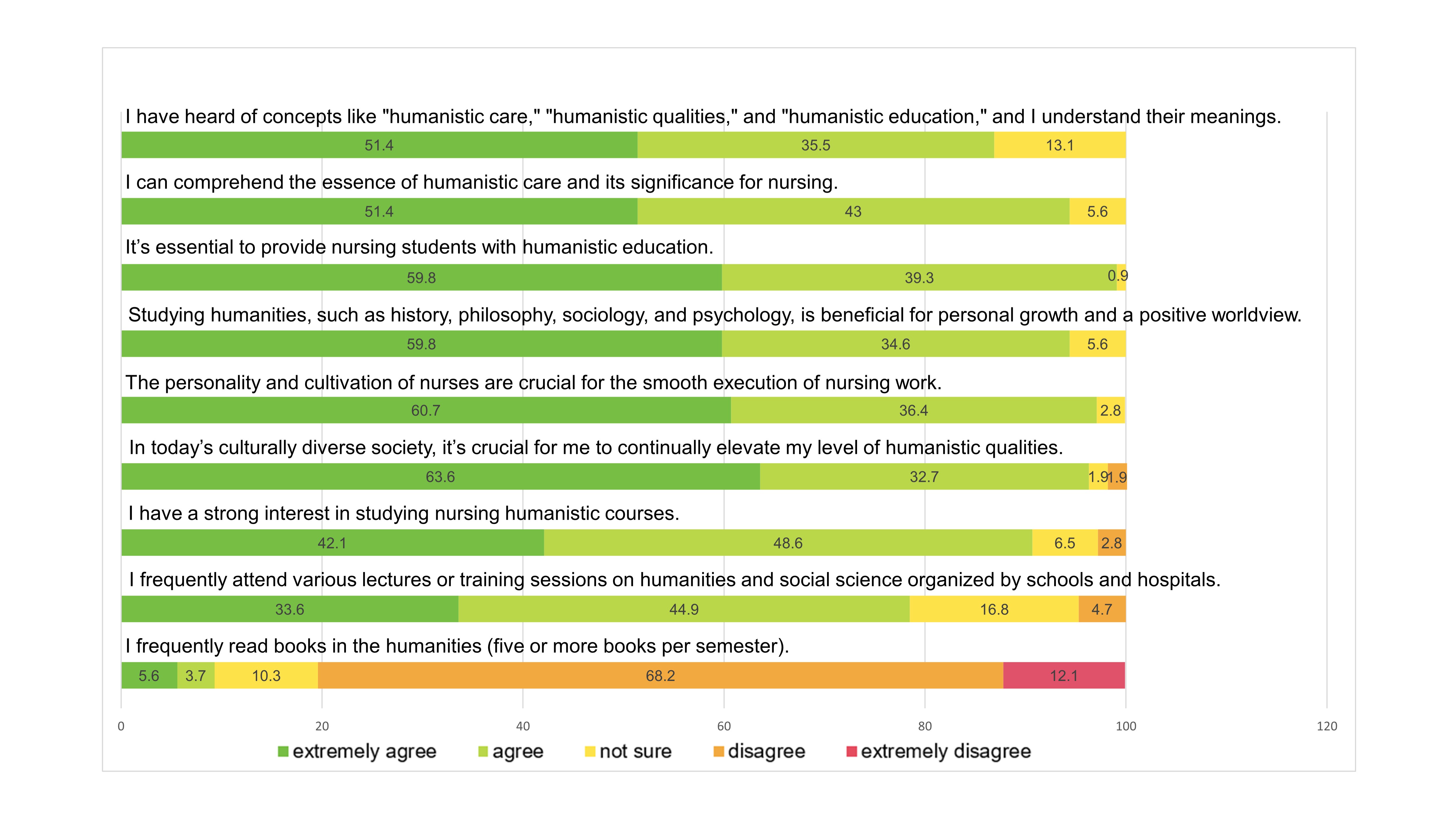

Supplement: Supplementary file 2 — Supplementary Material 2 [file 12909_2024_5377_MOESM2_ESM.jpg]

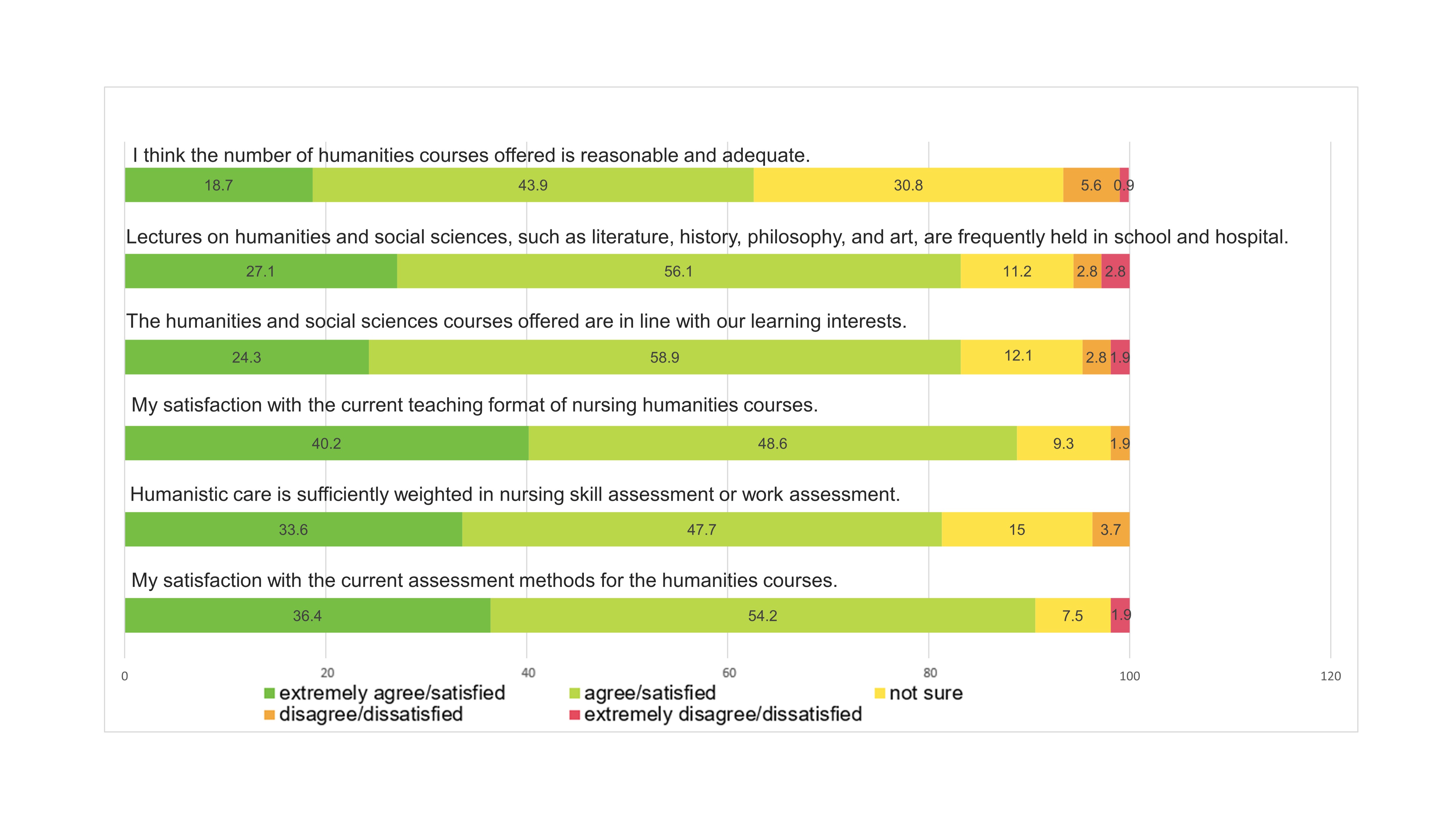

Supplement: Supplementary file 3 — Supplementary Material 3 [file 12909_2024_5377_MOESM3_ESM.jpg]

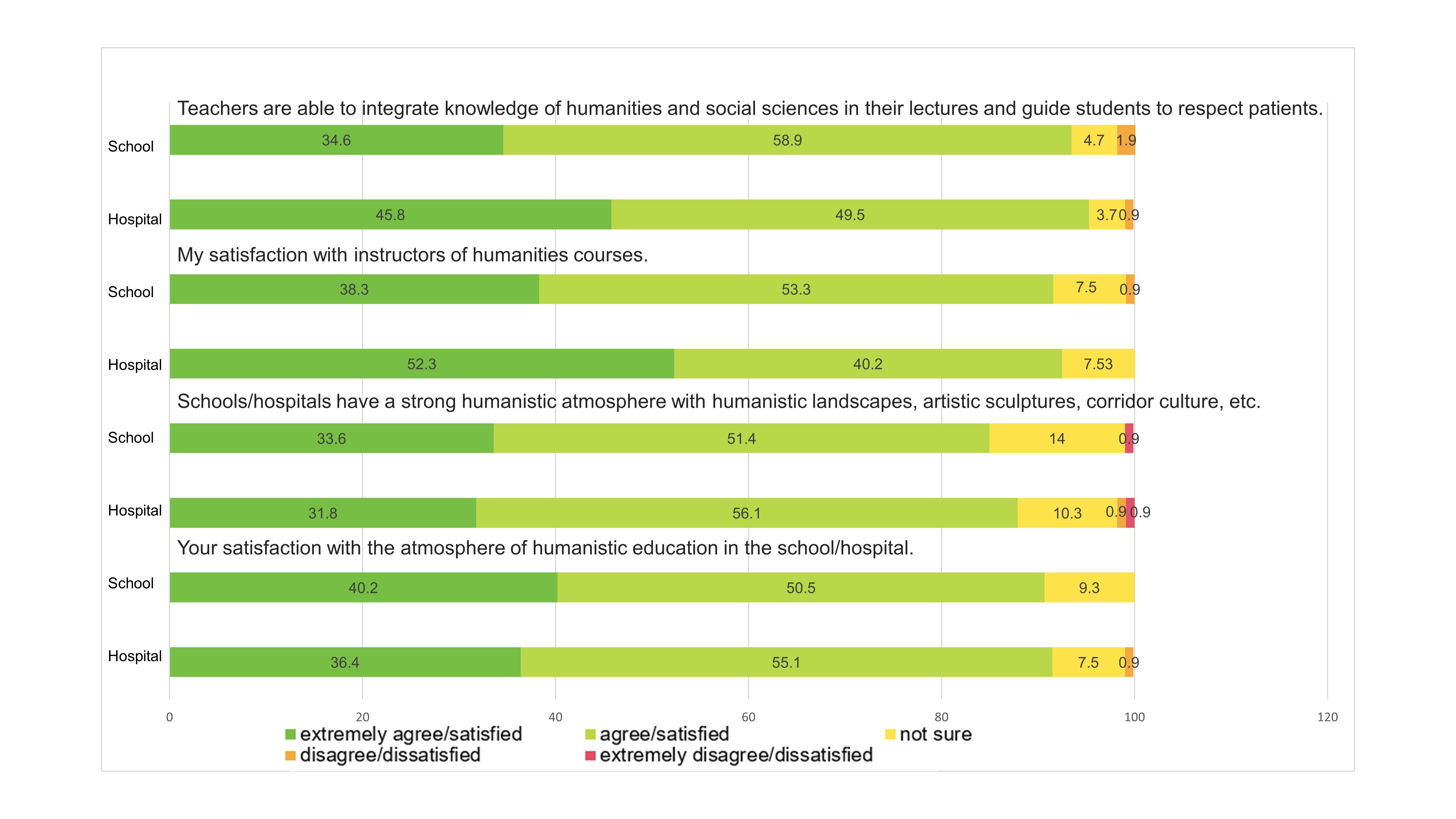

Supplement: Supplementary file 4 — Supplementary Material 4 [file 12909_2024_5377_MOESM4_ESM.jpg]
